# The *MDM4* SNP34091 (rs4245739) C-allele is associated with increased risk of ovarian—but not endometrial cancer

**DOI:** 10.1007/s13277-016-4940-2

**Published:** 2016-02-11

**Authors:** Liv B. Gansmo, Merete Bjørnslett, Mari Kyllesø Halle, Helga B. Salvesen, Anne Dørum, Einar Birkeland, Kristian Hveem, Pål Romundstad, Lars Vatten, Per Eystein Lønning, Stian Knappskog

**Affiliations:** 1 Section of Oncology, Department of Clinical Science, University of Bergen, Bergen, Norway; 2 Department of Oncology, Haukeland University Hospital, Bergen, Norway; 3 Department of Molecular Oncology, Institute for Cancer Research, Oslo University Hospital, Norwegian Radium Hospital, Oslo, Norway; 4 Faculty of Medicine, University of Oslo, Oslo, Norway; 5 Department of Gynecology and Obstetrics, Haukeland University Hospital, Bergen, Norway; 6 Center for Cancer Biomarkers CCBIO, Department of Clinical Science, University of Bergen, Bergen, Norway; 7 Department of Gynecologic Oncology, Oslo University Hospital, Norwegian Radium Hospital, Oslo, Norway; 8 Department of Public Health, Faculty of Medicine, Norwegian University of Science and Technology, Trondheim, Norway

**Keywords:** MDM4, SNP34091, Cancer risk, Ovarian cancer, Endometrial cancer

## Abstract

The MDM4 protein (also known as MDMX or HDMX) is a negative regulator of p53, not only by direct interaction but also through its interaction with MDM2. Further, *MDM4* overexpression and amplification have been observed in several cancer forms. Recently, a single nucleotide polymorphism (SNP) in the 3’ untranslated region of the *MDM4* gene, SNP34091A > C (rs4245739) was reported to alter *MDM4* messenger RNA (mRNA) stability by modulating a microRNA binding site, thereby leading to decreased MDM4 levels. In this case-control study, we aimed to evaluate the possible association between *MDM4* SNP34091 status and cancer risk by comparing the genotype frequencies in large hospital-based cohorts of endometrial- (*n* = 1404) and ovarian (*n* = 1385) cancer patients with healthy female controls (*n* = 1870). Genotype frequencies were compared by odds ratio (OR) estimates and Fisher exact tests. We found that individuals harboring the *MDM4* SNP34091AC/CC genotypes had a significantly elevated risk for serous ovarian cancer (SOC) in general and high-grade serous ovarian cancer (HGSOC) in particular (SOC: OR = 1.18., 95 % CI = 1.01–1.39; HGSOC: OR = 1.25, CI = 1.02–1.53). No association between SNP34091 genotypes and endometrial cancer risk was observed. Our data indicate the *MDM4* SNP34091AC/CC genotypes to be associated with an elevated risk for SOC and in particular the HGSOC type.

**Electronic supplementary material:**

The online version of this article (doi:10.1007/s13277-016-4940-2) contains supplementary material, which is available to authorized users.

## Introduction

Maintaining the correct levels of p53 is imperative to cell survival and normal tissue homeostasis, and thus, the p53 protein plays a pivotal role in cancer biology [1]. The protein product of the murine double minute 2 gene, *MDM2*, and its homologue *MDM4* (also referred to as *MDMX* or *HDMX*) are known to be the major negative regulators of p53 [2]. While both MDM2 and MDM4 inhibits p53 by direct binding and masking of its transactivation domain [3–6], only MDM2 possesses E3 ubiquitin ligase activity and may downregulate p53 by targeting it for ubiquitin-proteasome-dependent degradation [7–9]. However, heterodimerization with MDM4 enhances MDM2’s E3 ligase activity towards p53 [10, 11]. Taken together, these data suggest that elevated levels of MDM4 can prevent p53-mediated tumor suppression. In line with this, the *MDM4* gene has been found amplified and overexpressed in several cancer forms (reviewed in [12]), and studies in transgenic mice have shown that overexpression of Mdm4 induced spontaneous tumor formation and accelerated tumorigenesis [13].

In the last decades, several single nucleotide polymorphisms in the *MDM2* [14, 15] as well as the *MDM4* [16–18] loci have been associated with elevated or reduced cancer risk, although data are at variance. Recently, a single nucleotide polymorphism (SNP) in the *MDM4* 3’ untranslated region, *MDM4* SNP34091A > C (rs4245739) was reported to affect *MDM4* messenger RNA (mRNA) stability and protein levels [19, 20]. The SNP34091C variant creates a functional target site for hsa-miR-191, and both ovarian and prostate cancer cells harboring the C- allele displayed reduced MDM4 mRNA and protein levels [19, 20].

Conflicting evidence has linked rs4245739 genotypes to breast cancer risk. Thus, while case-control studies suggested the SNP34091C-allele to be associated with a reduced risk for breast cancer in general [17, 21] and among individuals carrying a *MDM2* SNP309GG genotype in particular [17], recent genome-wide association studies (GWAS) have reported the C-allele to be associated with an increased risk for estrogen receptor (ER) negative and, in particular, triple-negative breast cancer [22–24]. Regarding other malignancies, the C-allele has been associated with a reduced risk of non-Hodgkin lymphoma [25], esophageal squamous cell carcinoma [26], and prostate cancer [27] but not with risk of cancer of the lung or colon [17].

In this study, we assessed the impact of *MDM4* SNP34091 status on the risk of ovarian and endometrial cancer in large hospital-based sample sets.

## Materials and methods

### Study population

In this study, we successfully genotyped the *MDM4* SNP34091A > C (rs4245739) status in endometrial cancer samples (*n* = 1404) obtained from patients treated at Haukeland University Hospital during 2001–2009 [28] and from patients included in the MoMaTEC (Molecular Markers in Treatment of Endometrial Cancer) study between 2007–2010 [29]. Further, we genotyped ovarian cancer samples (*n* = 1385) from patients treated at the Oslo University Hospital Radium Hospital in the period between 1993 and 2011 [15]. Notably, patients with known mutations related to hereditary ovarian cancer (*BRCA1* or *BRCA2*) were excluded from the analysis.

For the endometrial cancer samples we had histological and FIGO status for 1321 and 1238 patients, respectively. As for the ovarian cancer, the grade of differentiation (high-grade serous ovarian cancer (HGSOC), low-grade serous ovarian cancer (LGSOC), clear cell ovarian cancer, endometrioid ovarian cancer, and mucinous ovarian cancer) was available for 1071 patients.

In order to evaluate OR for ovarian and endometrial cancer related to *MDM4* SNP34091 status, we compared our findings to the SNP status among 1870 healthy controls. These were the female fraction of a sample set of 3747 healthy individuals, previously genotyped [17], and they were all obtained from the population-based Cohort of Norway (CONOR) study [30].

### *MDM4* SNP34091 genotyping

All samples were genotyped for *MDM4* SNP34091 status using a custom LightSNiP assay (TIB MOLBIOL Syntheselabor GmbH, Berlin, Germany) on a LightCycler 480 II instrument (Roche, Basel, Switzerland) as previously described in detail [17].

### Statistical analysis

Potential deviations from Hardy-Weinberg equilibrium were assessed by calculating the expected genotype distribution based on the observed allele frequencies and comparing the output with the observed genotype distribution using chi-square tests.

Potential associations between *MDM4* SNP34091 and risk of ovarian and endometrial cancer as well as cancer risk within different subgroups were estimated by calculating odds ratios (OR) with 95 % confidence intervals (CI) and by Fisher’s exact tests.

All statistical analyses were performed using the IBM SPSS 22 software (IBM Corp, Armonk, NY, USA). *p* values are given as two-sided and *p* values from Fisher’s exact tests are given as cumulative.

## Results

### Distribution of *MDM4* SNP34091

Among the 1870 healthy female controls previously genotyped [17], we recorded a minor allele frequency (MAF) of 0.27. Regarding the present analyses, *MDM4* SNP34091 status was successfully genotyped in 1385 ovarian and 1404 endometrial cancers cases.

The genotype frequencies were found to be in Hardy-Weinberg equilibrium (*p* > 0.8 for all comparisons). A comprehensive overview of the *MDM4* SNP34091 distribution in the healthy controls as well as the two cancer types analyzed is given in Table 1.

**Table 1:** *MDM4* SNP34091 distribution and cancer risk

Cases/	Genotype			OR (95 % CI)	Fisher’s	OR (95 % CI)	Fisher’s
controls	SNP34091 *n* (%)			SNP34091	exact test	SNP309	exact test
	AA	AC	CC	CC vs. AA + AC		CC + AC vs. AA	
Controls (females)	1021 (54.6)	703 (37.6)	146 (7.8)	1.00	–	1.00	–
Endometrial cancer	757 (53.9)	541 (38.5)	106 (7.6)	0.95 (0.74–1.25)	0.792	1.03 (0.90–1.18)	0.723
Ovarian cancer	716 (51.7)	564 (40.7)	105 (7.6)	0.97 (0.75–1.26)	0.842	1.12 (0.98–1.29)	0.102

### *MDM4* SNP34091 status and cancer risk in ovarian cancer

In order to estimate the potential impact of *MDM4* SNP34091 status on ovarian cancer risk, we compared the frequency of the *MDM4* SNP34091 genotypes among ovarian cancer patients (*n* = 1385) to healthy female controls (*n* = 1870). Although we observed no significant association between *MDM4* SNP34091 status and ovarian cancer risk, applying the dominant model (SNP34091CC + AC vs. AA) we observed a non-significant association with increased risk for ovarian cancer (OR = 1.12; 95 % CI = 0.98–1.29; Table 1).

**Fig. 1:**
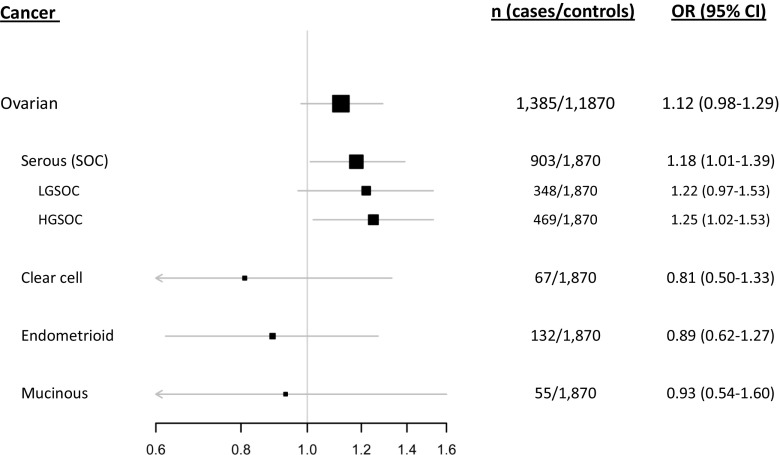
Impact of *MDM4* SNP34091 on ovarian cancer risk. Forest plot showing the effect of SNP34091 in the different histological ovarian cancer types as compared to healthy female controls. *LGSOC* low-grade serous ovarian cancer, *HGSOC* high-grade serous ovarian cancer

Following the observation of a potential association between the SNP34091CC/AC genotypes and increased risk of ovarian cancer in general, we performed separate analyses for the different subgroups of the disease with respect to histology class and grade of differentiation. By doing so, we observed a significant association between carriers of the SNP34091C allele (dominant model) and increased risk of serous ovarian cancer (OR = 1.18; 95 % CI = 1.01–1.39), but not any increased risk for clear cell, endometrioid, or mucinous ovarian cancers (Table 2, Fig. 1). Further, by stratifying serous ovarian carcinoma into the high-grade and low-grade type, we found that the increased risk conferred by the SNP34091C allele was highest in HGSOC (OR = 1.25; 95 % CI = 1.02–1.53; Table 2, Fig. 1).

**Table 2:** *MDM4* SNP34091 and cancer risk in the histological OC types

Cases/	Genotype			OR (95 % CI)	Fisher’s	OR (95 % CI)	Fisher’s
controls	SNP34091 *n* (%)			SNP34091	exact test	SNP309	exact test
	AA	AC	CC	CC vs. AA + AC		CC + AC vs. AA	
Controls (females)	1021 (54.6)	703 (37.6)	146 (7.8)	1.00	–	1.00	–
Serous (LG and HGSOC)	455 (50.4)	376 (41.6)	72 (8.0)	1.02 (0.76–1.37)	0.880	1.18 (1.01–1.39)	0.038
LGSOC	173 (49.7)	148 (42.5)	27 (7.8)	0.99 (0.65–1.52)	1.000	1.22 (0.97–1.53)	0.101
HGSOC	230 (49.0)	199 (42.4)	40 (8.5)	1.10 (0.76–1.59)	0.633	1.25 (1.02–1.53)	0.034
Clear cell (CC)	40 (59.7)	23 (34.3)	4 (6.0)	0.75 (0.27–2.09)	0.815	0.81 (0.50–1.33)	0.455
Endometrioid (E)	76 (57.6)	46 (34.9)	10 (7.6)	0.97 (0.50–1.89)	1.000	0.89 (0.62–1.27)	0.528
Mucinous (M)	31 (56.4)	20 (36.6)	4 (7.3)	0.92 (0.33–2.60)	1.000	0.93 (0.54–1.60)	0.891

### *MDM4* SNP34091 status and cancer risk in endometrial cancer

Comparing the genotypes of 1404 endometrial cancer patients to the 1870 healthy controls, no association between *MDM4* SNP34091 status and endometrial cancer were observed, either by applying the dominant or the recessive model (Table 1).

We further stratified the endometrial cancer patients according to histological and FIGO status; however, no association between SNP34091 genotypes and endometrial cancer risk was observed in any of the subgroups (data not shown).

### *MDM4* SNP34091 and interaction with *MDM2* SNP309

In the same sample set as analyzed in the present study, we have previously reported the genotypes for the two most widely studied functional SNPs in the *MDM2* gene (*MDM2* SNP285; rs117039649 and SNP309; rs2279744) [15, 28]. Since MDM4 and MDM2 act together in inhibiting the tumor suppressor function of p53, we investigated potential interactions/synergistic effects between *MDM4* SNP34091 and *MDM2* SNPs with respect to cancer risk.

Assessing ovarian cancer in general, we found a moderate synergistic effect of SNPs in the two genes. However, when restricting the analyses to HGSOC, we found particularly high risk of disease among individuals with the *MDM4* SNP34091C-allele and *MDM2* SNP309TT genotype (OR = 1.41; 95 % CI = 1.02–1.94; Supplementary Table S1).

In contrast, we found no synergistic effects between *MDM4* SNP34091 and *MDM2* SNPs with respect to endometrial cancer risk (data not shown).

### *MDM4* expression levels in ovarian and endometrial cell lines

Since we found an effect of *MDM4* SNP34901 in ovarian—but not endometrial cancer, we mined the publically available data set from the Broad-Novartis Cancer Cell Line Encyclopedia (CCLE–Broad Institute; www. broadinstitute.org/ccle/home). Comparing the available data, we found the average *MDM4* expression level among ovarian cancer cells (*n* = 51) to be significantly lower than endometrial cancer cells (*n* = 27; *p* = 0.003), indicating that ovarian cells may be more sensitive to subtle changes in MDM4 levels than endometrial cancer cells.

## Discussion

In this study, we examined the association between the *MDM4* SNP34091 status and ovarian- and endometrial cancer risk applying a case-control design. This is, to the best of our knowledge, the first case-control study estimating the effect of *MDM4* SNP34091 on risk for endometrial cancer and, although a small study found an increased risk for relapse and early onset of ovarian cancer among individuals carrying the SNP34091A allele [20], no study has evaluated *MDM4* SNP34091 status as a potential risk factor with respect to ovarian cancer.

In our overall analyses, we observed no association between *MDM4* SNP34091 status and the risk for endometrial cancer, but a non-significant association between the SNP34091C allele and an increased risk of ovarian cancer. Notably, individuals carrying the SNP34091C allele had significantly increased risk for developing serous ovarian cancer, and in particular tumors of the HGSOC type, compared to individuals harboring the SNP34091AA genotype. The risk was particularly high among individuals carrying the *MDM2* SNP309TT genotype. Although risk assessments are not directly comparable to survival analysis, our findings may seem somewhat contradictory to the report of the SNP34091A allele as a risk factor for recurrence and tumor-related death in ovarian cancer patients [20]. While the SNP34091A allele has been reported to confer higher MDM4 levels in ovarian [20] and prostate cancer cells [19], and the common assumption is that the oncogenic effect of high MDM4 levels is through the p53 pathway [31], it has been reported that over 90 % of all HGSOC have mutations in the *TP53* gene [32]. Thus, it may be that the effect of *MDM4* SNP34091 on ovarian cancer risk is mediated via additional pathways, other than p53.

While evidence linking SNP34091 status to breast cancer risk has been at variance [21–24], we recently found the *MDM4* SNP34091AC genotype to be associated with a reduced risk of breast cancer among individuals carrying the *MDM2* SNP309GG genotype [17], a genotype, in general, associated with elevated cancer risk [33, 34]. In the same study, no association between SNP34091 status and risk for cancer of the lung, colon, or prostate was observed [17]. Our findings in the present study are, however, in line with previous studies reporting SNP34091C to be associated with increased risk for triple-negative breast cancer [23, 24], a subclass of breast cancers sharing some mutational features with HGSOC [35]. The tissue-specific effects observed are also in line with the previously observed effect of the *MDM2* SNP285G > C; where the C-allele is proposed to reduce the risk for ovarian, endometrial, and breast cancer, but not cancer of the prostate, lung, or colon [15, 28, 36, 37]. Notably, among cell lines registered in Cancer Cell Line Encyclopedia (CCLE–Broad Institute), we found a lower average *MDM4* expression level among ovarian—than endometrial cancer cells. One may, therefore, speculate that ovarian cells in general are more sensitive than endometrial cells to subtle changes in the MDM4 levels, such as those induced by the different SNP34901 genotypes.

Previous candidate gene case-control studies assessing the effect of the SNP34091 on cancer susceptibility has been performed mainly in populations of Chinese ethnicity [21, 25, 26], and they have reported the SNP34091C-allele to be associated with a reduced risk of cancer. Notably, there is a substantial difference in the distribution of this SNP between Europeans and Asians with a MAF of 0.26 and 0.05, respectively [38]. This is also the case for the *MDM2* promoter SNPs, SNP309, and SNP285: while the SNP309G allele is associated with an increased cancer risk, predominantly, among individuals of Asian ancestry [33, 34], the SNP285C-allele, which is associated with reduced cancer risk, [15, 28, 36], is absent in Asians and may therefore have a confounding effect on SNP309 risk estimates performed in Caucasian populations [39]. Thus, the somewhat variable results regarding *MDM4* SNP34091 and cancer risk may also be explained by yet unknown functional SNP (s) that are in linkage disequilibrium (LD) with SNP34091.

A few years ago, Atwal and colleagues reported the *MDM4* haplotype diversity across ethnic populations. They found a much greater diversity among individuals of African American and Ashkenazi Jewish ancestry than Caucasians ancestry. Further, they reported the SNP rs1563828T allele, which is in LD with the SNP34091A allele among Caucasians, to be associated with early onset of both familial and sporadic ovarian cancer [16]. To the best of our knowledge, the biological effects of SNP rs1563828 have not been elucidated; thus, the possibility exist that it may be SNP34091, which is known to have a biological effect [19, 20], that contributes to the observed effect of SNP rs1563828 previously reported by Atwal and colleagues.

In conclusion, we find the *MDM4* SNP34091 to be associated with increased risk of SOC, in particular the HGSOC type, and in particular among individuals carrying the *MDM2* SNP309TT genotype. In contrast, no effect on endometrial cancer risk was recorded. Although the observed ORs are too low to argue for any clinical use of *MDM4* SNP status, further studies are warranted in order to reveal whether it could be a useful marker in any subgroup of cancers.

## Electronic supplementary material

Below is the link to the electronic supplementary material.

Supplementary Table S1 (DOCX 15 kb)
