# Supplementary material for: The MDM4 SNP34091 (rs4245739) C-allele is associated with increased risk of ovarian—but not endometrial cancer
Source: Tumour Biol. 2016 Feb 11;37(8):10697–702. doi: 10.1007/s13277-016-4940-2 (PMC4999457; doi:10.1007/s13277-016-4940-2)
Supplement: Supplementary file 1 — (DOCX 15 kb) [file 13277_2016_4940_MOESM1_ESM.docx]

| **Supplementary Table 1. *MDM4* SNP34091 and risk for HGSOC among *MDM2* SNP309** | | | | | | | | | | | |
| --- | --- | --- | --- | --- | --- | --- | --- | --- | --- | --- | --- |
| **Cases/** | **Genotype** | | |  | **OR (95% CI)** |  | **Fisher** |  | **OR (95% CI)** |  | **Fisher** |
| **controls** | **SNP34091 n (%)** | | |  | **SNP34091** |  | **exact** |  | **SNP309** |  | **exact** |
|  | **AA** | **AC** | **CC** |  | **CC vs. AA+AC** |  |  |  | **CC+AC vs. AA** |  |  |
| **309TT** |  |  |  |  |  |  |  |  |  |  |  |
| **HGSOC** | 88 (47.1) | 81 (43.3) | 18 (9.6) |  | 1.30 (0.75-2.27) |  | 0.365 |  | 1.41 (1.02-1.94) |  | 0.040 |
| **309TG** |  |  |  |  |  |  |  |  |  |  |  |
| **HGSOC** | 103 (49.5) | 89 (42.8) | 16 (7.7) |  | 1.09 (0.62-1.94) |  | 0.776 |  | 1.13 (0.84-1.53) |  | 0.441 |
| **309GG** |  |  |  |  |  |  |  |  |  |  |  |
| **HGSOC** | 39 (52.7) | 29 (39.2) | 6 (8.1) |  | 0.71 (0.28-1.79) |  | 0.665 |  | 1.27 (0.76-2.14) |  | 0.423 |
